# High body mass index is a risk factor for difficult deep sedation in percutaneous mitral valve repair

**DOI:** 10.1371/journal.pone.0190590

**Published:** 2018-01-05

**Authors:** Katharina Hellhammer, Shazia Afzal, Renate Tigges, Maximilian Spieker, Tienush Rassaf, Tobias Zeus, Ralf Westenfeld, Malte Kelm, Patrick Horn

**Affiliations:** 1 University Hospital Düsseldorf, Medical Faculty, Dept. of Medicine, Division of Cardiology, Pulmonology and Vascular Medicine, Düsseldorf, Germany; 2 University Hospital Essen, Medical Faculty, Dept. of Medicine, Division of Cardiology and Vascular Medicine, Essen, Germany; University of Bern, University Hospital Bern, SWITZERLAND

## Abstract

**Background:**

The safety and efficacy of deep sedation (DS) in MitraClip^®^ procedures have been shown previously. However, clinical experience demonstrates that in some patients DS is difficult to achieve. We hypothesize that some patient characteristics can predict difficult DS.

**Methods:**

We prospectively analysed 69 patients undergoing MitraClip^®^ procedures using DS. Application of DS was graded as simple (group 1) or difficult (group 2) depending on a cumulative score based on one point for each of the following criteria: decrease in oxygen saturation, retention of carbon dioxide, disruptive body movements, and the need for catecholamines. Patients with one point or less were classified as group 1, and patients with two or more points were classified as group 2.

**Results:**

In 58 of 69 patients (84.1%), the performance of DS was simple, while in 11 patients (15.9%), DS was difficult to achieve. Patients with difficult DS were characterized by a higher body mass index (33.7 ± 6.0 kg/m^2^ vs. 26.1 ± 4.1; p = 0.001), younger age (67 ± 13 years vs. 75 ± 13 years; p = 0.044), and reduced left ventricular ejection fraction (36% ± 10 vs. 45% ± 14; p = 0.051) and presented more often with an obstructive sleep apnoea syndrome (6.9% vs. 45.5%; p = 0.003). In the multivariate analysis, body mass index was an independent predictor of difficult DS. Using a body mass index of 31 kg/m^2^ as a cut-off value, the sensitivity of predicting difficult DS was 73%, and the specificity was 88%. Using a body mass index of 35 kg/m^2^ as a cut-off value, the specificity increased to 97%, with a sensitivity of 36%.

**Conclusion:**

In patients with a higher body mass index who undergo MitraClip^®^ procedures, DS might be difficult to perform.

## Introduction

Percutaneous mitral valve repair (PMVR) has evolved into an effective therapeutic option in high-risk patients with severe mitral regurgitation (MR) and is routinely performed using general anaesthesia (GA). In general, GA is associated with a mortality risk of 0.03 deaths per 1000 patients [1]. However the risk of GA might be higher in patients considered for PMVR compared to the average population as they are characterized by advanced age with severely impaired left ventricular function and various comorbidities. With the advent of minimally invasive therapeutic options for the treatment of valvular heart disease for most of these severely morbid patients, the concept of the use of deep sedation (DS) as opposed to GA has emerged. Previous studies have shown that the MitraClip^®^ procedure can be performed safely and efficiently using DS [2–5]. Furthermore, we have shown that MitraClip^®^ procedures using DS require less preparation time in the catheter laboratory than procedures performed using GA [2]. Another study demonstrated a significantly shorter stay in the intensive care unit (ICU) for patients in which MitraClip^®^ procedures were performed with DS [4]. However, clinical experience reveals that DS remains difficult to perform at least in some patients. As the procedure requires transoesophageal guidance, a certain level of DS is needed to assure optimal conditions for this challenging intervention. This must be considered when performing DS to avoid haemodynamic and respiratory impairment, as well as agitation and body movement. We hypothesize that some patient characteristics can predict difficult DS in patients undergoing PMVR with the MitraClip^®^ system.

## Materials and methods

### Study design

From July 2016 to December 2016, we prospectively investigated 69 consecutive patients who underwent PMVR with the MitraClip^®^ system using DS. In our institution, the MitraClip^®^ procedure has been routinely performed using DS since 2011. Preprocedural assessment of MR followed the current guidelines for the management of valvular heart disease [6]. All cases were discussed by the local heart team, and patients with high surgical risk or who were unsuitable for surgery were denied surgical valve repair. All patients provided written informed consent for data acquisition and analysis. The study was conducted in accordance with the ethical guidelines of the 1975 Declaration of Helsinki, and the institutional Ethics Committee of the Heinrich Heine University approved the study protocol (approval number 4497R). All data were included in a registry, which is listed at www.clinicaltrials.gov (NCT02033811).

### Performance of deep sedation

DS was performed using continuous administration of propofol (2%) through a central venous catheter placed in the left femoral vein over a 7F sheath. All patients received local anaesthesia with 10 ml of 0.2% lidocaine administered subcutaneously at the femoral access site. Catecholamines (norepinephrine at a dose of 5 mg/50 ml) and saline were available if needed. Haemodynamic monitoring and measurements were obtained through a radial arterial catheter. All patients received 2–3 mg midazolam 30 min prior to the procedure as well as 0.5 mg of atropine when starting the procedure. In addition to haemodynamic and respiratory monitoring, the level of sedation was assessed and monitored using the Richmond Agitation-Sedation Scale (RASS) with the goal of a score of minus 3 to ensure an adequate level of sedation for a safe procedure. We started the sedation by administering a propofol bolus that was adjusted to the patient’s actual body weight. As patients were denied for surgery due to high operative risk and being categorized as American Society of Anesthesiologists (ASA) class III, we used a dose of 0.5 mg/kg of 2% propofol. Half of the calculated bolus was initially given followed by a partial dose of the remaining amount within five minutes while monitoring haemodynamic and respiratory response as well as the depth of sedation according to the RASS score. If the desired sedation level was not achieved after the initial bolus, we administered further propofol boluses (0.25 mg/kg) in one-min intervals until the sedation level was reached. When the desired sedation level was achieved, we continued sedation by continuous administration of propofol with a dosage of 1.5 mg/kg/h according to the RASS score and haemodynamic and respiratory monitoring. In the case of disruptive body movements, we administered a single bolus of 0.25–0.5 mg/kg.

All patients received oxygen through a nasal cannula with a baseline flow of 2 l/min when starting the procedure. Equipment for endotracheal ventilation was prepared for every procedure. All patients were monitored by continuous measurement of peripheral oxygen saturation to detect a decrease in oxygen. Blood gas analysis was performed every 15 min or in shorter periods if needed to monitor respiratory status (carbon dioxide, pH and oxygen levels). As we did not use capnography, patient ventilation effort and airflow were assessed by monitoring the respiratory rate, watching the breathing pattern, feeling the chest movement and lung auscultation. The oxygen flow was increased depending on peripheral oxygen saturation (with a goal of > 95%). The patient’s head was positioned in a light head-tilt position. In case of a relevant decrease of oxygen (< 92%) or retention of carbon dioxide (> 60 mmHg with a pH < 7.25), a head-tilt/chin-lift manoeuvre was performed. Furthermore, propofol administration was reduced, and a Wendl tube was inserted if the head-tilt/chin-lift manoeuvre was not sufficient.

Patients were monitored by a cardiologist with more than 12 months of training in intensive care medicine. An anaesthesiology team was on site for all procedures.

After the procedure was completed, all patients were transferred directly to the ICU for 24 h of surveillance.

### Assessment of deep sedation

No general criteria or assessment scores have been defined to assess difficult DS in cardiac interventions. We therefore implemented a protocol with standardized criteria defining difficult DS in patients undergoing MitraClip^®^ procedures based on our clinical experience in DS for cardiovascular interventions and derived from patients undergoing an endoscopic gastrointestinal intervention [7]. We defined and assessed criteria associated with difficult DS as follows (Table 1): 1.) need for catecholamines (norepinephrine in a dosage of 0.1 μg/kg/min for at least 10 min, 2.) persistent decrease in oxygen saturation < 85% requiring airway manoeuvres and oxygen insufflation > 5 l/min, 3.) retention of carbon dioxide > 60 mmHg with a pH < 7.25 requiring airway manoeuvres and 4.) more than five disruptive body movements.

**Table 1:** Criteria for evaluation of complexity of deep sedation and classification of deep sedation according to score.

Criteria	Assessment	Score
Disruptive body movement	More than 5	1 point
Carbon dioxide > 60 mmHg and pH < 7.25	Yes/No	1 point
Decrease in oxygen < 85% with need for oxygen > 5 l/min	Yes/No	1 point
Need for catecholamines	Yes/No	1 point
**Score**		
0–1 point	Group 1 (simple)	
> = 2 points	Group 2 (complex)	

Airway manoeuvres were classified a priori as chin lift manoeuvres and manoeuvres to improve upper airway or nasopharyngeal airway patency for optimal airflow. A disruptive body movement was defined as a movement of the patient leading to interruption of the procedure and the need for an additional sedation bolus.

One point was given for each criterion fulfilled, with a maximum score of 4 points. Patients with ≤ 1 point were classified to group 1, which was considered simple DS. Patients with ≥ 2 points were assigned to group 2, which was considered difficult DS. In the case of conversion to GA, the patient was classified as group 2.

### Safety and efficacy assessment

Periprocedural data and in-hospital adverse events were reported for all patients. The safety of the procedure was evaluated by assessment of bleeding or vascular complications, rates of postinterventional pneumonia and acute kidney injury, and occurrence of a major adverse cardiac or cerebrovascular event (MACCE). The definition of a MACCE included death, ST-elevation myocardial infarction, stroke and procedure-related re-operation. Minor and major vascular complications, minor and major bleeding complications and acute kidney injury were defined according to the standardized endpoint definitions of the Valve Academic Research Consortium II [8].

The efficiency of the procedure was assessed by procedural success, defined as reduction of initial MR of at least one grade, procedure time, device time and radiation time. Procedure time was defined as the time from vascular puncture until closure of the femoral access. Device time was defined as the time from steerable guide catheter placement in the intra-atrial septum until the time the clip delivery system was retracted into the steerable guide catheter.

### Percutaneous mitral valve repair

Technical details of the MitraClip® system and the procedure have been described in detail elsewhere [9]. The procedure was performed using DS as described and was guided by transoesophageal echocardiography (TEE) and fluoroscopy.

### Statistical analysis

Continuous variables were assessed for a normal distribution using the Kolmogorov-Smirnov-test and are expressed as the means ± standard deviation. For small sample sizes, we performed non-parametric tests (Mann-Whitney U Test) to compare means between the two groups. For categorical variables, frequencies are expressed as percentages and were compared using a chi-square test or a Fisher’s exact test. A two-tailed p-value < 0.05 was considered statistically significant for all tests. Data analysis was performed with SPSS^®^ Statistics 22 (IBM^®^, Armonk, NY, USA). Multivariate logistic regression analysis was used to correlate variables with a difficult or simple DS. Patient variables included in the multivariable model consisted of those with a p-value < 0.1 in the correlation analysis (Spearman’s rank correlation). A receiver operating characteristic analysis was performed to determine the cut-off values.

## Results

In 58 of 69 patients (84.1%), DS was simple, and in 11 patients (15.9%), DS was difficult to perform. One patient (1.5%) had to be converted to GA due to respiratory depression. The patient characteristics are listed in Table 2.

**Table 2:** Clinical characteristics of patients grouped according to complexity of deep sedation.

	Group 1 (n = 58)	Group 2 (n = 11)	p-value
Age (years)	75 ± 13	67 ± 13	**0.044**
Male	34.8% (24)	54.4% (6)	0.513
LogES (%)	27.5 ± 16.6	18.4 ± 9.3	0.07
Score	0.7 ± 0.8	2.3 ± 0.7	**0.001**
BMI (kg/m^2^)	26.1 ± 4.1	33.7 ± 6.0	**0.001**
ASA class	2.8 ± 0.4	2.8 ± 0.4	0.851
COPD	26% (15)	54.5% (6)	0.077
OSAS	6.9% (4)	45.5% (5)	**0.003**
FEV1 (l)	1.6 ± 0.6	1.9 ± 0.7	0.247
VC (l)	2.4 ± 0.7	2.8 ± 0.8	0.116
GFR (ml/min)	48 ± 20	59 ± 20	0.112
Bilirubin (mg/dl)	0.7 ± 0.3	0.9 ± 0.5	0.189
Ejection fraction (%)	45 ± 14	36 ± 10	0.051
TAPSE (mm)	17 ± 6	18 ± 4	0.532
PAsys (mmHg	45 ± 15	48 ± 12	0.394
CI (l min-1)	2.0 ± 0.4	2.0 ± 0.3	0.948
Previous surgery on neck/head	0% (0)	0% (0)	1.0
Previous complications during anesthesia	0% (0)	0% (0)	1.0

ASA = American Society of Anethesiologists; BMI = body mass index; CI = cardiac index; COPD = chronic obstructive pulmonary disease; FEV1 = forced exspiration volume in one second; GFR = glomerular filtration rate; LogES = logistic EuroSCORE; OSAS = obstructive sleep apnoea syndrome; PAsys = systolic pulmonary artery pressure; TAPSE = tricuspid annular plane systolic excursion; VC = vital capacity

Patients with difficult DS were younger (67 ± 13 years vs. 75 ± 13 years; p = 0.044), had a higher body mass index (BMI) (33.7 ± 6.0 kg/m^2^ vs. 26.1 ± 4.1 kg/m^2^; p = 0.001), had a lower left ventricular ejection fraction (36 ± 10% vs. 45 ± 14%; p = 0.051) and presented more often with obstructive sleep apnoea syndrome (6.9% vs. 45.5%; p = 0.003) than patients with simple DS. Further patient characteristics are listed in the supporting information file (S1 File). All patients with difficult DS had a decrease in oxygen saturation. The need for catecholamines was observed in 4 of 11 patients, 4 of 11 patients had a relevant retention of carbon dioxide, and 6 of 11 patients had disruptive body movements (Table 3).

**Table 3:** Characterization of patients with a complex deep sedation.

	Need for catecholamines	Decrease in oxygen < 85%	Retention of carbon dioxide > 60 mmHg	Disruptive body movement
Patient 1		+	+	
Patient 2		+		+
Patient 3		+		+
Patient 4	+	+		+
Patient 5	+	+		+
Patient 6	+	+		
Patient 7		+	+	
Patient 8	+	+	+	
Patient 9		+		+
Patient 10		+		+
Patient 11		+	+	
**In total**	**4**	**11**	**4**	**6**

In a multivariate regression analysis (including age, BMI, left ventricular function and the presence of obstructive sleep apnoea syndrome), only higher BMI was found to be an independent predictor for difficult DS (Table 4).

**Table 4:** Multivariate logistic regression.

	Complex deep sedation		
	B	p-value	95% CI
BMI	1.438	0.006	1.109–1.836
Age	0.940	0.270	0.841–1.049
OSAS	1.680	0.666	0.160–17.644
LV function	0.969	0.302	0.891–1.036

BMI = Body mass index; OSAS = obstructive sleep apnoea syndrome; LV = left ventricular

In our study, no patient with a BMI < 27 kg/m^2^ was characterized by difficult DS. Using a BMI of 31 kg/m^2^ as a cut-off value, the sensitivity to predict difficult DS was 73% and the specificity was 88%. Using a BMI of 35 kg/m^2^ as a cut-off value, the specificity increased to 97%, with a sensitivity of 36%.

The mean procedure time, device time and fluoroscopy time were similar in both groups. Procedural success was achieved in 100% of the patients, independent of classification of DS. The mean number of implanted clips was higher in patients with difficult DS (1.5 ± 0.5 vs.1.1 ± 0.3; p = 0.039). Analysis of the mean propofol dosage administered during the procedure showed a greater need for propofol in patients with difficult DS (2.5 ± 2.9 mg/kg/h vs. 12.0 ± 0.6 mg/kg/h; p = 0.561). The mean dosage of norepinephrine was significantly greater in patients with difficult DS (0.08 ± 0.01 μg/kg/min vs. 0.13 ± 0.03 μg/kg/min; p = 0.023) (Table 5).

**Table 5:** Procedural data and in-hospital course of patients grouped according to complexity of deep sedation.

	Group 1 (n = 58)	Group 2 (n = 11)	p-value
Successful clip implantation	100% (58)	100% (11)	1.0
Fluoroscopy time (min)	31 ± 16.8	34 ± 11.3	0.726
Device time (min)	61 ± 33	58 ± 33.0	0.888
Number of Clips (n)	1.5 ± 0.5	1.1 ± 0.3	**0.039**
Conversion to surgery	0% (0)	0% (0)	1.0
Conversion to GA	0% (0)	9% (1)	0.159
Mean propofol dose (mg/kg/h)	2.0 ± 0.6	2.5 ± 2.9	0.561
Mean weight-based dose of norepinephrine (ug/kg/min)	0.08 ± 0.01	0.13 ± 0.03	**0.023**
MACCE	0% (0)	0% (0)	1.0
In-hospital mortality	0% (0)	0% (0)	1.0
Bleeding complications	3.4% (2)	0% (0)	1.0
Vascular complications	1.7% (1)	0% (0)	1.0
Pneumonia	5.2% (3)	18% (2)	0.177
Sepsis	0% (0)	0% (0)	1.0
Acute kidney injury	3.4% (2)	9% (1)	0.411

GA = general anaesthesia; MACCE = major cardiac and cerebrovascular events

No in-hospital deaths occurred. Post procedural pneumonia occurred in three of the patients with simple DS (5.2%) and in 2 of 11 (18.2%) patients with difficult DS (p = 0.177). Bleeding complications (3.4% vs. 0%; p = 1.0), vascular complications (1.7% vs. 0%; p = 1.0), MACCE (0% vs. 0%), occurrence of sepsis (0% vs. 0%) and acute kidney injury (3.4% vs. 9%; p = 0.411) did not differ between the two groups (Table 5).

## Discussion

The major finding of our study is that in patients undergoing MitraClip^®^ procedures, a higher BMI is associated with difficult DS.

Our study confirms the results of previous studies that demonstrated the safety and efficacy of MitraClip^®^ procedures using DS [2; 3; 10]. Only one patient (1.5%) had to be converted to GA due to respiratory depression, which is a low conversion rate and consistent with previous reports [2; 5]. Procedural success was achieved in 100% of the patients irrespective the difficulty of DS. No in-hospital deaths occurred. We previously reported in a larger study that mortality did not differ between MitraClip^®^ procedures using DS and procedures using GA [2]. These mortality rates are comparable to those reported for MitraClip^®^ procedures using GA, which have been reported as 2.5% in the TRAMI registry [11] and 4.8% in the EVEREST II high-risk study [12].

Adverse event rates of pneumonia, renal failure, stroke or vascular complications were low and have been described in equal numbers in MitraClip^®^ procedures performed under GA or DS [2; 4]. In a previous report, the rate of pneumonia in patients undergoing DS for MitraClip^®^ procedures was 6.7% [4]. In our study, pneumonia occurred in 7.2% of the patients. However, a non-significant trend was observed of a higher rate of pneumonia in patients with complex DS. This may be related to the difficult airway management and a potential higher risk of micro- and macro-aspiration, which are concerns in patients undergoing MitraClip^®^ procedures using DS. Larger studies are needed to evaluate this aspect.

Taken together, the MitraClip^®^ procedure performed using DS is as safe and effective as MitraClip^®^ implantation performed using GA. Furthermore, the preparation time in the catheter laboratory [2] and the stay in the intensive care unit (ICU) was shorter when MitraClip^®^ procedures were performed with DS [4].

To our best knowledge, this is the first study that characterizes the quality of DS performance and identifies parameters of difficult DS. Although the level of experience with DS plays a prominent role, we attempted to identify difficult DS based on objective parameters in this study. The challenge of DS is to maintain an adequate level of sedation, which assures an optimal procedural condition to avoid respiratory failure and haemodynamic compromise. In previous reports of upper intestinal endoscopy procedures using DS, an ASA class ≥ 3 was the most powerful predictor of airway manoeuvres and sedation-related complications [13–15]. However, patients undergoing MitraClip^®^ procedures are nearly all characterized as ASA Class ≥ 3; thus, the ASA score does not help to identify patients at risk for a difficult DS in our setting.

Patients undergoing PMVR are mostly elderly and denied for surgery due to severe comorbidities. In our study, patients with difficult DS presented with a higher BMI, worsened left ventricular function, more frequent obstructive sleep apnoea syndrome, and surprisingly, they were also of younger age. The pharmacokinetics of elderly patients differ from those of younger patients due to age-dependent physiological changes. Higher plasma levels and prolonged elimination require decreased sedation and vice versa, and thus younger patients may require higher dosages. This may provoke an over-dosage in younger patients and, in the case of an obese patient, desaturation and retention of carbon dioxide. In our study, patients with difficult DS had a greater need for propofol. As propofol leads to central nervous system depression and vasodilatation, dose-dependent hypotension is one of the most frequent complications, particularly when given as a bolus. The relationship between propofol dosage and the need for catecholamines therefore must be considered when comparing catecholamine dosages. However, patients with difficult DS had a significantly higher need for catecholamines.

Obesity is known to be a risk factor for hypoxemia in patients undergoing DS for upper intestinal endoscopy [16]. The pharmacokinetics of drugs may be unpredictable in obese patients, and the volume of distribution is increased for lipid soluble agents, such as propofol and fentanyl [17], possibly resulting in the need for a higher dose of these agents to reach the target level of sedation and resulting in prolonged elimination. In addition, airway management can be challenging in these patients. Experienced staff and expertise in DS are mandatory and are provided in highly experienced centres performing high numbers of procedures using DS (including transcatheter aortic valve implantation and left atrial appendage occlusion). In addition, sedation in this risk group should be managed by professionals trained in advanced airway management (trained anaesthesia professionals or experienced ICU physicians).

### Study limitations

Our study has several limitations. First, this was a single centre study with a limited number of patients. The findings must be confirmed by larger prospective trials. Second, the classification of DS was based on parameters that we assumed to reflect difficult DS. As mentioned, no exact parameters have been defined to describe difficult DS in MitraClip^®^ procedures. These parameters were defined based on clinical experience in DS for cardiovascular interventions and derived from patients undergoing an endoscopic gastrointestinal intervention [7].

### Conclusion

In patients with a high BMI (> 31 kg/m^2^), MitraClip^®^ procedures might be difficult to perform using DS. Depending on the experience of the centre in performing DS, patients with a BMI > 35 kg/m^2^ should be considered to receive the procedure under GA.

## Supporting information

S1 File Patient characteristics. BMI = body mass index; CI = cardiac index; COPD = chronic obstructive pulmonary disease; FEV1 = forced expiratory volume in one second; GFR = glomerular filtration rate;LogES = logistic EuroSCORE; OSAS = obstructive sleep apnoea syndrome; PAsys = systolic pulmonary artery pressure; TAPSE = tricuspid annular plane systolic excursion; VC = vital capacity.
(PDF)

## Abbreviations

ASA: American Society of Anaesthesiologists
BMI: Body mass index
CI: Cardiac index
COPD: Chronic obstructive pulmonary disease
DS: Deep sedation
FEV1: Forced expiratory volume in one second
GA: General anaesthesia
GFR: Glomerular filtration rate
ICU: Intensive Care Unit
LogES: Logistic EuroSCORE
MACCE: Major adverse cardiac and cerebrovascular, event
OSAS: Obstructive sleep apnoea syndrome
PAsys: Systolic pulmonary artery pressure
PMVR: Percutaneous mitral valve repair
RASS: Richmond Agitation-Sedation Score
TAPSE: Tricuspid annular plane systolic excursion
TEE: Transoesophageal echocardiography
VC: Vital capacity
