# Supplementary material for: High body mass index is a risk factor for difficult deep sedation in percutaneous mitral valve repair
Source: PLoS One. 2018 Jan 5;13(1):e0190590. doi: 10.1371/journal.pone.0190590 (PMC5755851; doi:10.1371/journal.pone.0190590)
Supplement: S1 File — BMI = body mass index; CI = cardiac index; COPD = chronic obstructive pulmonary disease; FEV1 = forced expiratory volume in one second; GFR = glomerular filtration rate;LogES = logistic EuroSCORE; OSAS = obstructive sleep apnoea syndrome; PAsys = systolic pulmonary artery pressure; TAPSE = tricuspid annular plane systolic excursion; VC = vital capacity. (PDF) [file pone.0190590.s001.pdf]

| Age | Sex (Female/male) | BMI | GFR | COPD | Ejection fraction (%) | CI |
|---|---|---|---|---|---|---|
| 54 | 0 | 16 | 55 | 0 | 32 | 1.5 |
| 64 | 1 | 19 | 64 | 0 | 30 | 2 |
| 68 | 1 | 21 | 21 | 1 | 29 | 2 |
| 73 | 1 | 21 | 21 | 1 | 50 | 1.97 |
| 79 | 1 | 21 | 12 | 0 | 45 | 2.2 |
| 77 | 1 | 21 | 23 | 1 | 20 | 2 |
| 74 | 1 | 21 | 38 | 0 | 62 | 2.1 |
| 77 | 0 | 22 | 47 | 0 | 20 | 1.7 |
| 82 | 1 | 22 | 54 | 0 | 60 | 2.1 |
| 0 | 1 | 22 | 35 | 0 | 43 | 2.2 |
| 70 | 1 | 22.4 | 51 | 0 | 40 | 1.97 |
| 84 | 0 | 22.5 | 86 | 1 | 58 | 2.43 |
| 79 | 0 | 22.7 | 37 | 0 | 28 | 1.94 |
| 80 | 1 | 23 | 94 | 0 | 60 | 1.92 |
| 69 | 0 | 23 | 64 | 0 | 42 | 2.3 |
| 68 | 1 | 23 | 56 | 0 | 45 | 2 |
| 83 | 1 | 23 | 36 | 0 | 53 | 2 |
| 74 | 1 | 24 | 76 | 0 | 42 | 1.76 |
| 91 | 1 | 24 | 22 | 0 | 60 | 1.72 |
| 80 | 1 | 24 | 46 | 1 | 60 | 1.96 |
| 82 | 0 | 24 | 65 | 0 | 30 | 2 |
| 86 | 0 | 24 | 47 | 0 | 47 | 1.77 |
| 70 | 0 | 24 | 88 | 0 | 26 | 2.2 |
| 58 | 0 | 24.3 | 84 | 0 | 33 | 2.36 |
| 91 | 1 | 25.7 | 16 | 0 | 58 | 2.5 |
| 76 | 1 | 26 | 63 | 1 | 53 | 1.85 |
| 78 | 1 | 26 | 47 | 0 | 40 | 2.2 |
| 56 | 0 | 26 | 75 | 1 | 36 | 2 |
| 68 | 0 | 26 | 72 | 1 | 27 | 1.45 |
| 83 | 1 | 26 | 37 | 0 | 44 | 2.5 |
| 84 | 0 | 26.2 | 48 | 1 | 40 | 2.44 |
| 84 | 1 | 26.2 | 57 | 0 | 45 | 1.88 |
| 67 | 1 | 27 | 61 | 1 | 60 | 2 |
| 89 | 1 | 27 | 50 | 0 | 60 | 1.83 |
| 77 | 0 | 27 | 34 | 0 | 53 | 2.9 |
| 86 | 1 | 27 | 35 | 1 | 60 | 2.2 |
| 60 | 0 | 27 | 63 | 0 | 15 | 2 |
| 73 | 1 | 27 | 30 | 0 | 60 | 1.6 |
| 78 | 0 | 27 | 78 | 1 | 49 | 1.87 |
| 74 | 0 | 27 | 20 | 1 | 44 | 1.96 |
| 56 | 0 | 27 | 61 | 1 | 15 | 1.6 |
| 78 | 0 | 27.5 | 48 | 0 | 35 | 1.23 |
| 85 | 0 | 28 | 31 | 0 | 40 | 2.6 |
| 79 | 0 | 28 | 46 | 1 | 45 | 1.99 |
| 78 | 0 | 28.6 | 55 | 0 | 35 | 2.66 |
| 65 | 1 | 29 | 26 | 0 | 45 | 1.8 |
| 86 | 0 | 29 | 17 | 0 | 45 | 2.7 |
| 79 | 0 | 29.4 | 49 | 0 | 30 | 1.7 |
| 59 | 0 | 29.9 | 67 | 0 | 19 | 2.7 |

| | | | | | | |
|---|---|---|---|---|---|---|
| 85 | 1 | 30 | 46 | 0 | 62 | 1.88 |
| 78 | 1 | 30 | 66 | 0 | 39 | 2.2 |
| 81 | 1 | 30 | 58 | 0 | 62 | 2 |
| 82 | 1 | 30 | 55 | 0 | 60 | 1.49 |
| 79 | 0 | 30.1 | 49 | 0 | 30 | 1.5 |
| 85 | 1 | 31 | 42 | 0 | 60 | 1.43 |
| 70 | 0 | 31 | 10 | 1 | 45 | 1.7 |
| 80 | 1 | 31 | 38 | 0 | 60 | 2.6 |
| 70 | 1 | 31 | 76 | 0 | 31 | 2.1 |
| 78 | 0 | 31 | 53 | 0 | 35 | 2.5 |
| 59 | 0 | 32 | 56 | 1 | 22 | 2.4 |
| 52 | 0 | 33 | 100 | 0 | 30 | 2.04 |
| 82 | 1 | 34 | 43 | 0 | 60 | 2 |
| 80 | 1 | 34 | 34 | 1 | 60 | 1.9 |
| 89 | 1 | 35 | 46 | 0 | 78 | 2.6 |
| 88 | 1 | 35.4 | 47 | 0 | 40 | 1.6 |
| 80 | 0 | 36 | 25 | 1 | 20 | 2.3 |
| 51 | 1 | 41 | 59 | 1 | 40 | 2 |
| 81 | 1 | 41 | 56 | 1 | 50 | 2.3 |
| 58 | 1 | 44 | 73 | 0 | 40 | 1.7 |

| LogES | PAsys | Bilirubine | TAPSE | FEV1 | VC | OSAS |
|---|---|---|---|---|---|---|
| 30 | 21 | 0.92 | 10 | 1.07 | 1.37 | 0 |
| 23 | 31 | 0.87 | 19 | 1.4 | 1.9 | 0 |
| 27 | 64 | 0.28 | 23 | 1.39 | 2.97 | 0 |
| 16 | 52 | 0.58 | 17 | 1,5 | 2.77 | 0 |
| 47 | 68 | 0.81 | 46 | 1.5 | 2.5 | 0 |
| 41 | 55 | 0.89 | 13 | 1.6 | 2.5 | 0 |
| 5.2 | 42 | 0.67 | 17 | 1.54 | 1.75 | 0 |
| 12.6 | 60 | 0.6 | 9 | 1.64 | 2.34 | 0 |
| 16.8 | 51 | 0.39 | 20 | 1.6 | 2.5 | 0 |
| 20 | 33 | 0.8 | 19 | 1.3 | 3.1 | 0 |
| 25 | 58 | 0.3 | 15 | 2.2 | 1.9 | 0 |
| 10 | 46 | 0.4 | 24 | 0.8 | 2 | 0 |
| 14.7 | 43 | 0.3 | 13 | 1.7 | 3.5 | 0 |
| 7.9 | 40 | 0.94 | 24 | 1.6 | 2.5 | 0 |
| 35 | 40 | 0.75 | 19 | 2.87 | 3.55 | 0 |
| 25 | 45 | 0.25 | 17 | 2.37 | 3.14 | 0 |
| 30.4 | 37 | 1.29 | 16 | 1.04 | 1.35 | 0 |
| 14 | 41 | 1.27 | 21 | 1.01 | 1.43 | 0 |
| 20 | 48 | 0.26 | 13 | 0.94 | 2.42 | 0 |
| 40 | 36 | 0.38 | 17 | 0.77 | 1.3 | 0 |
| 9.7 | 45 | 0.6 | 25 | 0.96 | 1.49 | 0 |
| 34 | 44 | 1.1 | 15 | 1.69 | 2.04 | 0 |
| 25 | 38 | 0.98 | 19 | 2.2 | 3 | 0 |
| 16 | 44 | 0.3 | 15 | 1.9 | 2.9 | 0 |
| 19 | 45 | 1.1 | 12 | 1.4 | 1.9 | 0 |
| 25 | 45 | 0.5 | 19 | 0.7 | 2.3 | 1 |
| 11 | 55 | 0.6 | 12 | 2.2 | 3.1 | 0 |
| 15.6 | 28 | 0.86 | 23 | 0.84 | 2.52 | 1 |
| 12 | 31 | 0.77 | 24 | 1.63 | 2.66 | 0 |
| 66 | 43 | 0.55 | 16 | 1.6 | 2.5 | 0 |
| 34 | 80 | 0.8 | 15 | 0.9 | 2.2 | 0 |
| 44.5 | 63 | 0.5 | 16 | 1.8 | 2.9 | 0 |
| 28 | 51 | 0.54 | 18 | 1.6 | 2.5 | 0 |
| 36.9 | 60 | 0.42 | 22 | 1.6 | 2.5 | 0 |
| 10.3 | 37 | 0.87 | 20 | 3.22 | 4.2 | 0 |
| 52 | 58 | 0.7 | 14 | 0.71 | 0.95 | 0 |
| 17 | 26 | 1.2 | 12 | 2.59 | 3.96 | 0 |
| 25 | 24 | 2.2 | 7 | 1.2 | 1.9 | 0 |
| 42 | 29 | 0.7 | 19 | 1.6 | 2.5 | 0 |
| 35.8 | 62 | 0.66 | 18 | 0.96 | 1.63 | 0 |
| 24 | 28 | 1.3 | 14 | 1.5 | 2.8 | 0 |
| 49 | 84 | 0.8 | 13 | 1.5 | 2.5 | 0 |
| 33 | 34 | 0.85 | 12 | 1.6 | 2.5 | 0 |
| 12.6 | 50 | 0.79 | 27 | 2.48 | 4.01 | 1 |
| 23 | 36 | 0.5 | 14 | 2 | 2.8 | 0 |
| 9.4 | 62 | 0.24 | 17 | 1.66 | 2.39 | 0 |
| 58 | 53 | 0.22 | 18 | 1.6 | 2.5 | 0 |
| 36 | 60 | 0.5 | 14 | 1.9 | 2.7 | 1 |
| 18 | 35 | 0.4 | 12 | 2.4 | 4 | 0 |

| | | | | | | |
|---|---|---|---|---|---|---|
| 25 | 36 | 0.81 | 13 | 1.83 | 1.01 | 0 |
| 36.5 | 66 | 0.6 | 21 | 1.62 | 2.17 | 0 |
| 71 | 47 | 0.93 | 21 | 1.3 | 1.9 | 0 |
| 15 | 46 | 0.79 | 22 | 1.02 | 1.51 | 0 |
| 40,5 | 60 | 0.4 | 13 | 1.4 | 2.2 | 0 |
| 3,9 | 45 | 0.44 | 19 | 0.98 | 1.44 | 0 |
| 49 | 75 | 0.77 | 17 | 2.02 | 2.5 | 0 |
| 15 | 21 | 0.22 | 20 | 1.67 | 2.28 | 0 |
| 11.4 | 53 | 0.71 | 24 | 2.61 | 3 | 0 |
| 12 | 60 | 2.2 | 14 | 2.5 | 3 | 0 |
| 7 | 43 | 0.58 | 17 | 1.9 | 3.1 | 1 |
| 13.9 | 28 | 1.1 | 17 | 3.13 | 4.25 | 1 |
| 5.1 | 38 | 0.39 | 23 | 3.5 | 1.4 | 0 |
| 39 | 10 | 0.87 | 14 | 1.4 | 1.92 | 0 |
| 14 | 48 | 0.64 | 17 | 1.6 | 2.1 | 0 |
| 25 | 50 | 0.6 | 16 | 1.1 | 2.2 | 0 |
| 74 | 24 | 0.42 | 13 | 1.69 | 2.27 | 1 |
| 11.4 | 54 | 0.57 | 15 | 1.24 | 1.87 | 0 |
| 31 | 42 | 0.45 | 20 | 1.9 | 3.1 | 1 |
| 18 | 63 | 0.8 | 17 | 1.5 | 2.6 | 1 |
